# The RING E3 ligase RLIM drives oxidative stress-induced stem cell dysfunction through MDM2-p53 signaling

**DOI:** 10.1016/j.jbc.2026.111391

**Published:** 2026-03-20

**Authors:** Xiaoyue Li, Caiqi Shen, Yajie Li, Lijun Wu, Jia Gao, Dong Zhu, Dong Dong, Feifei Chen, Peisheng Jin

**Affiliations:** 1 Department of Plastic Surgery, Affiliated Hospital of Xuzhou Medical University, Xuzhou, China; 2 Xuzhou Medical University, Xuzhou, China; 3 Jiangsu Center for the Collaboration and Innovation of Cancer Biotherapy, Cancer Institute, Xuzhou Medical University, Xuzhou, Jiangsu, China; 4 Center for Cleft Lip and Palate Treatment, Plastic Surgery Hospital, Chinese Academy of Medical Sciences and Peking Union Medical College, Beijing, China

**Keywords:** autophagy, cellular senescence, diabetes wound healing, human umbilical cord mesenchymal stem cells (hUCMSCs), ubiquitination

## Abstract

Chronic diabetic ulcers present a persistent challenge due to delayed wound healing. At the wound site, oxidative stress impairs stem cell survival and differentiation, accelerates senescence, and impairs autophagy. RLIM was identified as a critical regulator in human umbilical cord mesenchymal stem cells (hUCMSCs), where oxidative stress-induced RLIM upregulation leads to MDM2 degradation and stabilization of p53. Functionally, RLIM upregulation under oxidative stress inhibited autophagy, induced cellular senescence, and significantly impaired angiogenesis, cell migration, and immunomodulatory functions, ultimately hindering diabetic wound healing *in vivo*. These results highlight the RLIM-MDM2-p53 signaling axis as a pivotal pathway governing stem cell senescence and function under oxidative stress, offering promising therapeutic targets to enhance stem cell-based approaches for diabetic wound repair.

Diabetes mellitus (DM) is a chronic metabolic disorder marked by persistent hyperglycemia, which has emerged as a significant global health issue (1). Among its most challenging complications is impaired wound healing, often resulting in chronic, non-healing ulcers that severely impact patients' quality of life and elevate risks of infection, amputation, and mortality (2). Despite advancements in glycemic control, infection management, and wound care, effective therapies that fundamentally enhance tissue regeneration in diabetic wounds remain scarce. Stem cell-based therapies, particularly those utilizing hUCMSCs, have gained attention as promising strategies due to their abundant availability, low immunogenicity, and intrinsic ability to promote angiogenesis, modulate immune responses, and facilitate tissue repair (3, 4). However, the diabetic wound microenvironment, characterized by persistent oxidative stress, severely compromises stem cell survival, differentiation, and function, thus limiting their therapeutic efficacy (5).

Cellular senescence is a central mechanism contributing to stem cell dysfunction and reduced regenerative potential in the diabetic context (6). Autophagy, a highly conserved lysosomal degradation process, plays a pivotal role in maintaining cellular homeostasis and delaying senescence (7). Our previous research demonstrated that autophagy protects against oxidative stress-induced apoptosis, underscoring its role in stem cell survival (8). Moderate autophagy eliminates damaged organelles and proteins, alleviates oxidative damage, delays senescence, and sustains stem cell function. In contrast, autophagy impairment accelerates senescence and weakens regenerative capacity. Existing evidence suggests that in the diabetic microenvironment, oxidative stress-induced abnormal autophagy is closely linked to stem cell senescence; however, the precise molecular mechanisms remain inadequately understood (9, 10). Further studies from our group revealed that stem cells migrating into diabetic wounds exhibit significantly increased senescence and markedly reduced autophagy (11). Building upon these findings, this study further investigated the interplay between senescence and stem cell regulation under oxidative stress.

The MDM2-p53 signaling axis is a crucial pathway that governs the cell cycle, apoptosis, and senescence, with its stability regulated by various ubiquitination modifications (12). However, its role in the diabetic microenvironment has not been fully elucidated. Previous research from our team demonstrated that E3 ubiquitin ligases are essential in diabetic wound healing by regulating the degradation of key signaling molecules (13). In the present study, RLIM, an E3 ubiquitin ligase, was significantly upregulated under oxidative stress conditions. RLIM promoted the ubiquitination and degradation of MDM2, resulting in enhanced p53 stability, which ultimately triggered premature senescence and inhibited autophagy in hUCMSCs. Moreover, TRIM36, an upstream regulatory factor, mediated RLIM degradation through direct interaction, thereby partially mitigating the senescence effects induced by RLIM.

In conclusion, this study provides the first comprehensive insight into the central role of the RLIM-MDM2-p53 signaling pathway in regulating hUCMSCs senescence and autophagy. It identifies the key molecular mechanisms through which oxidative stress in the diabetic microenvironment leads to stem cell dysfunction, offering new theoretical perspectives and potential targets for optimizing hUCMSCs-based therapies for diabetic wound treatment.

## Results

### Oxidative stress induces ROS accumulation and promotes senescence in hUCMSCs

**Figure 1:**
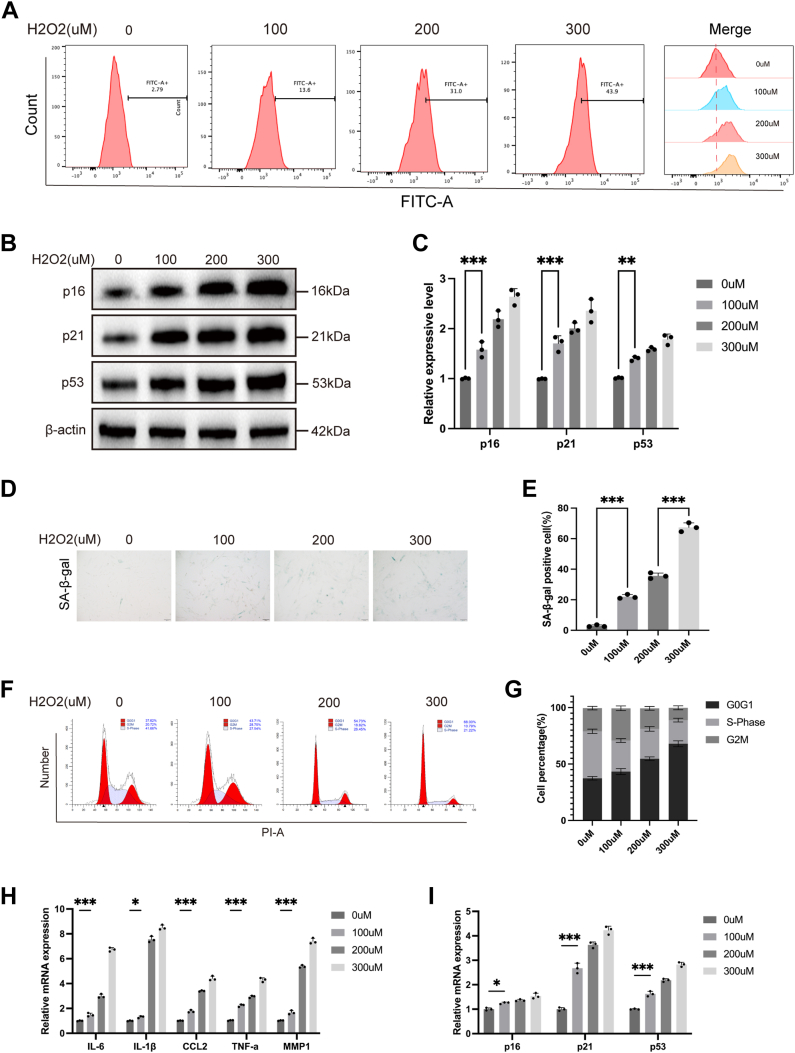
**Establishment of an oxidative stress-induced senescence model in hUCMSCs.***A*, flow cytometry analysis of intracellular ROS levels in hUCMSCs treated with various concentrations of H_2_O_2_ (0, 100, 200, 300 μM) (n = 3). *B and C*, Western blot analysis and quantification of senescence-associated proteins (p16, p21, and p53) (n = 3). *D and E*, representative images and quantification of SA-β-gal staining indicating the proportion of senescent cells (n = 3). Scale bar = 100 μm. *F and G*, cell cycle analysis of hUCMSCs by flow cytometry. Data are representative of three independent experiments (n = 3). *H and I*, qPCR analysis of senescence-related gene expression levels (n = 3). All data are representative of at least three independent experiments and presented as mean ± standard deviation (SD). Statistical analysis was performed using one-way ANOVA followed by Sidak’s multiple comparison test to compare differences among multiple groups. ∗*p* < 0.05, ∗∗*p* < 0.01 and ∗∗∗*p* < 0.001.

To examine the impact of oxidative stress on hUCMSCs senescence, cells were t exposed to varying concentrations of H_2_O_2_ (0, 100, 200, 300 μM). Intracellular ROS levels were measured using the fluorescent probe DCFH-DA and analyzed by flow cytometry. The results (Fig. 1*A*) demonstrated that as H_2_O_2_ concentrations increased, intracellular ROS levels progressively accumulated, confirming the successful induction of oxidative stress (14). Western blot analysis revealed a significant upregulation of senescence-associated proteins, including p16, p21, and p53, corresponding to elevated H_2_O_2_ concentrations (15) (Fig. 1, *B* and *C*). Following the confirmed induction of oxidative stress by H_2_O_2_, cellular senescence was also assessed using SA-β-gal staining, a method that detects the activity of a lysosomal enzyme, which is a well-established biomarker of senescent cells. SA-β-gal staining revealed a notable increase in the proportion of positive cells in the high-concentration H_2_O_2_ group, indicating that ROS accumulation effectively promoted senescence in hUCMSCs (Fig. 1, *D* and *E*). Cell cycle analysis further indicated that oxidative stress treatment caused G1-phase arrest in hUCMSCs, resulting in a decline in cell proliferation potential (Fig. 1, *F* and *G*). Additionally, qPCR analysis revealed a significant increase in the transcription levels of multiple senescence-related genes (Fig. 1, *H* and *I*). Autophagy levels were also assessed, and results consistently demonstrated a gradual decline in autophagic activity as H_2_O_2_ concentration increased (Fig. S1, *A*–*F*). In summary, our study establishes that oxidative stress induced by H_2_O_2_ accelerates the senescence of hUCMSCs, while concomitantly inhibiting autophagy.

### RLIM is upregulated in oxidative stress-treated hUCMSCs and inhibits autophagy while promoting senescence

**Figure 2:**
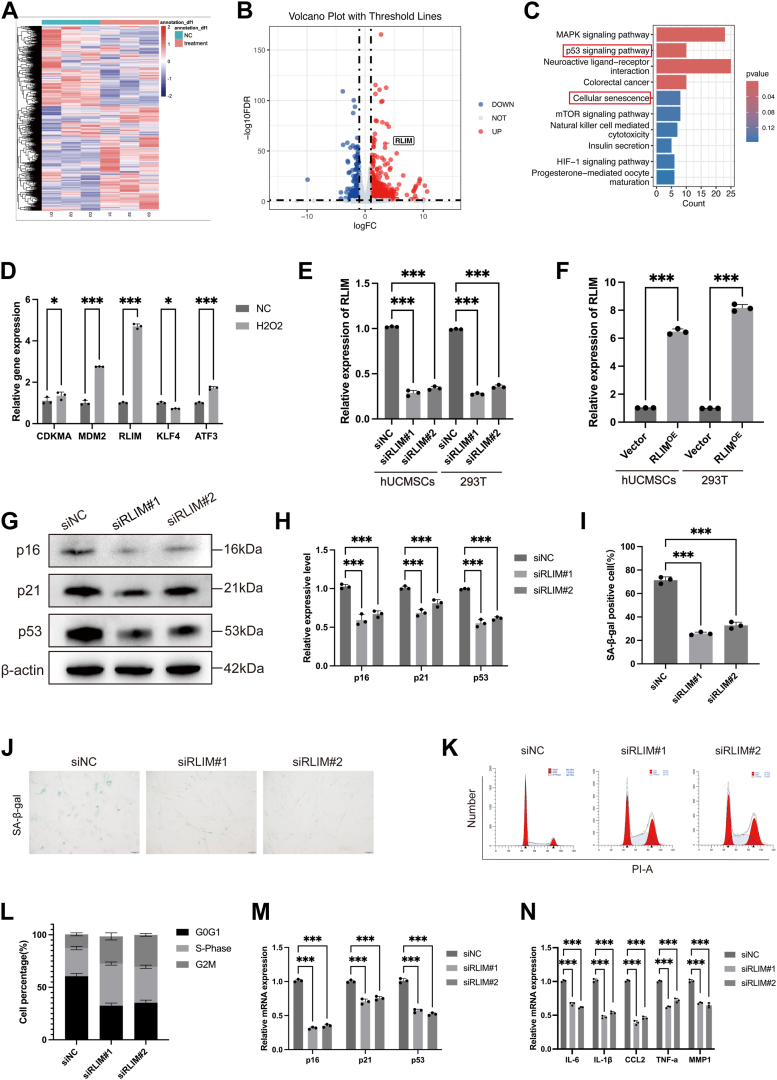
**Oxidative stress promotes RLIM expression and enhances senescence in hUCMSCs.***A*, RNA-seq workflow comparing hUCMSCs under oxidative stress and control conditions. *B*, Volcano plot showing marked RLIM upregulation in oxidative stress-treated cells. Statistical significance for differentially expressed genes (DEGs) in the volcano plot is defined by |log_2_ fold change (logFC)| > 1 and *p*-value < 0.05. *C*, KEGG pathway analysis revealing enrichment in p53 signaling and senescence-related pathways. Pathway enrichment significance is determined by *p*-value < 0.05. *D*, qRT-PCR validation of selected differentially expressed genes (n = 3). *E and F*, Western blot validation of RLIM overexpression and knockdown efficiency in hUCMSCs and 293T cells (n = 3). *G and H*, Western blot analysis and quantification of senescence-related proteins (p16, p21, and p53) in RLIM-knockdown hUCMSCs (n = 3). *I and J*, representative images and quantification of SA-β-gal staining showing the proportion of senescent cells in RLIM-knockdown hUCMSCs (n = 3). Scale bar = 100 μm. *K and L*, cell cycle analysis of RLIM-knockdown hUCMSCs by flow cytometry (n = 3). *M and N*, qPCR analysis of senescence-related gene expression levels in RLIM-knockdown hUCMSCs (n = 3). All data are representative of at least three independent experiments and shown as mean ± standard deviation (SD). Statistical analysis was performed using Student’s *t* test for pairwise comparisons (control vs. oxidative stress-treated, siNC vs. siRLIM, Control vs. RLIM^OE^). Significance levels are indicated as ∗*p* < 0.05, ∗∗*p* < 0.01, and ∗∗∗*p* < 0.001.

To identify key regulatory factors involved in the functional impairment of hUCMSCs under oxidative stress, RNA sequencing was performed on hUCMSCs treated with H_2_O_2_ and control-treated cells (Fig. 2*A*). Among the differentially expressed genes, RLIM exhibited significant upregulated (Fig. 2*B*). KEGG pathway analysis revealed that the differentially expressed genes were notably enriched in the p53 signaling pathway and senescence-related pathways (Fig. 2*C*), suggesting that these pathways are integral to the regulatory mechanism. GSEA enrichment analysis further confirmed the significant enrichment of these pathways (Fig. S2*A*). qRT-PCR analysis of several highly differentially expressed genes revealed that RLIM showed the most significant change in expression levels (Fig. 2*D*). To further investigate the role of RLIM, cell models with controllable RLIM expression were developed by upregulating and downregulating RLIM levels (Fig. 2, *E* and *F*). For the loss-of-function studies, three distinct siRNAs targeting RLIM were screened, and siRLIM#1, which demonstrated the highest knockdown efficiency (Fig. S1, *G* and *H*), was selected and used in all subsequent experiments. To exclude off-target effects and ensure reliability, a second independent siRNA (siRLIM#2) was also employed to validate all key phenotypes. Under oxidative stress conditions, RLIM overexpression in hUCMSCs significantly inhibited autophagy (Fig. S3, *G*–*L*) and exacerbated the senescence phenotype (Fig. S2, *B*–*I*). Conversely, RLIM knockdown enhanced autophagic flux (Fig. S3, *A*–*F*) and reduced senescence (Fig. 2, *G*–*N*). RLIM knockdown resulted in enhanced autophagic activity, reduced senescence markers such as SA-β-Gal staining, a restored normal cell phenotype, and partial recovery of cell cycle progression and senescence-related gene expression.

### RLIM knockdown alleviates senescence of hUCMSCs induced by oxidative stress through activation of autophagy

**Figure 3:**
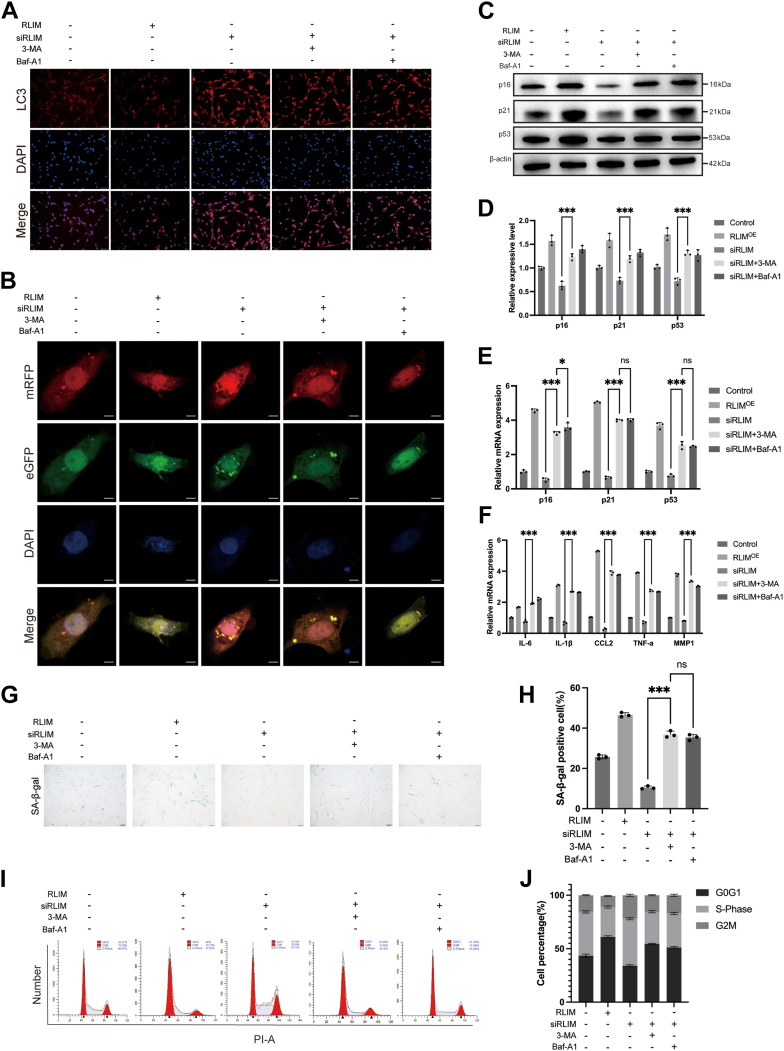
**RLIM knockdown promotes autophagy and inhibits cellular senescence in hUCMSCs.***A*, immunofluorescence staining of LC3 showing autophagic activity in RLIM knockdown cells treated with autophagy inhibitors 3-MA and Baf-1 (n = 3). Scale bar = 50 μm. *B*, confocal images of autophagosomes in adenovirus-infected hUCMSCs following RLIM knockdown and inhibitor treatment. Scale bar = 5 μm. *C and D*, Western blot analysis of p16, p21, and p53 expression after RLIM knockdown and inhibitor exposure (n = 3). *E and F*, qPCR analysis of senescence-related gene expression following RLIM knockdown and inhibitor treatment (n = 3). *G and H*, SA-β-gal staining of hUCMSCs after RLIM knockdown and inhibitor treatment (n = 3). Scale bar = 100 μm. (I, J) Flow cytometry analysis of cell cycle distribution in hUCMSCs after RLIM knockdown and inhibitor exposure (n = 3). All data are representative of at least three independent experiments and shown as mean ± standard deviation (SD). Statistical analysis was performed using one-way ANOVA followed by Sidak’s multiple comparison test for comparisons among multiple groups (Control, RLIM^OE^, siRLIM, siRLIM + 3-MA, siRLIM + Baf-A1). Significance levels are indicated as ∗*p* < 0.05, ∗∗*p* < 0.01, and ∗∗∗*p* < 0.001, “ns” indicates no significant difference (*p* ≥ 0.05).

To assess the role of RLIM-mediated autophagy regulation in hUCMSCs senescence, autophagy inhibitors 3-methyladenine (3-MA, 5 mM) and bafilomycin A1 (Baf-1, 100 nM) were applied to RLIM-knockdown hUCMSCs, targeting the early and late stages of autophagy, respectively. The results demonstrated that RLIM knockdown significantly enhanced autophagic activity, as evidenced by the increased accumulation of autophagosomes and autophagic flux (Fig. 3, *A* and *B*). Autophagic flux was monitored using an mRFP-GFP-LC3 adenovirus, where both GFP and RFP signals indicate autophagosomes. GFP signal quenching upon fusion with lysosomes left only the RFP signal, enabling clear compartment distinction. The increased yellow (GFP + RFP) and red (RFP-only) puncta confirmed that RLIM knockdown promoted both autophagosome accumulation and autophagic flux, providing real-time visualization of autophagy levels. This increase in autophagy activity was associated with a reduction in senescence markers, including decreased expression of P16, P21, and P53, lower mRNA levels of senescence-related genes, reduced SA-β-Gal staining, and partial restoration of cell cycle progression (Fig. 3, *C*–*J*). Notably, treatment with the autophagy inhibitors (3-MA and Baf-1) reversed these protective effects, indicating that the anti-senescence function of RLIM knockdown is largely mediated by autophagy. These findings suggest that RLIM promotes oxidative stress-induced stem cell senescence by inhibiting autophagy, while its knockdown mitigates senescence and preserves cellular function and autophagic homeostasis through autophagy activation. Therefore, RLIM assumes a pivotal regulatory role in the senescence process by modulating autophagy levels under conditions of oxidative stress.

### RLIM targets MDM2 ubiquitination and degradation

**Figure 4:**
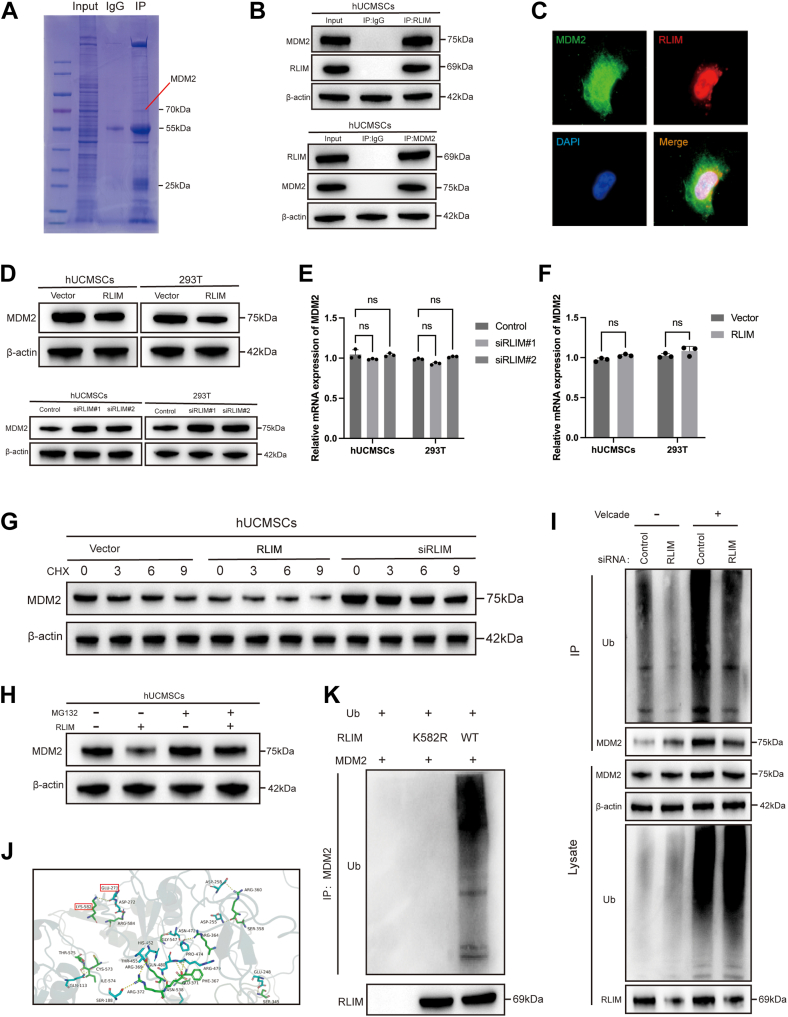
**RLIM interacts with and promotes ubiquitin-mediated degradation of MDM2.***A*, immunoprecipitation (IP) combined with mass spectrometry (MS) to identify RLIM-interacting proteins in hUCMSCs. *B*, co-immunoprecipitation (Co-IP) confirming direct binding between RLIM and MDM2 in hUCMSCs and 293T cells. *C*, dual immunofluorescence staining showing co-localization of RLIM and MDM2 in hUCMSCs. Scale bar = 5 μm. *D*, Western blot analysis illustrating changes in MDM2 protein expression following RLIM overexpression or knockdown (n = 3). *E and F*, qRT-PCR analysis showing no significant change in MDM2 mRNA levels upon RLIM overexpression or knockdown (n = 3). *G*, CHX chase assay demonstrating altered MDM2 protein stability in RLIM-modulated cells (n = 3). *H*, Western blot showing partial rescue of MDM2 degradation by the proteasome inhibitor MG132 in RLIM-overexpressing cells (n = 3). *I*, ubiquitination assay indicating decreased polyubiquitination of MDM2 upon RLIM knockdown (n = 3). *J*, molecular docking analysis predicting the interaction between RLIM and MDM2, highlighting strong binding at residues GLU-271 and LYS-582. *K*, *in vitro* ubiquitination assay showing that wild-type RLIM, but not mutant RLIM, promotes MDM2 ubiquitination (n = 3). All data are representative of at least three independent experiments and shown as mean ± standard deviation (SD). Statistical analysis was performed using Student’s *t* test for pairwise comparisons (vector vs. RLIM^OE^/siRLIM, RLIM^OE^ vs. RLIM^OE^ + MG132, RLIM WT vs. RLIM K582 R). Significance levels are indicated as ∗*p* < 0.05, ∗∗*p* < 0.01, and ∗∗∗*p* < 0.001, “ns” indicates no significant difference (*p* ≥ 0.05).

RLIM, a classical E3 ubiquitin ligase, primarily exerts its biological functions by mediating ubiquitination and proteasomal degradation of downstream target proteins (16, 17). Previous studies have demonstrated that RLIM is regulated by other E3 ligases, such as TRIM28, and influences p53 stability in the context of lung tumorigenesis, affecting cell proliferation, migration, and survival (18). To explore the molecular mechanisms by which RLIM regulates hUCMSCs function under oxidative stress, particularly through its downstream targets, this study employed immunoprecipitation (IP) combined with mass spectrometry (MS) to identify interacting proteins (Fig. 4*A*). MS analysis identified MDM2 as a key interacting protein, with a high MS score. Co-immunoprecipitation (co-IP) experiments in hUCMSCs and 293T cells suggested a potential interaction between RLIM and MDM2, as evidenced by the specific enrichment of MDM2 in the anti-RLIM immunoprecipitates compared to the IgG control. The input and the immunoprecipitated RLIM itself are also shown to demonstrate the effectiveness of the IP (Figs. 4*B* and S4*A*). Additionally, dual immunofluorescence (IF) staining demonstrated clear co-localization of RLIM and MDM2 in hUCMSCs (Fig. 4*C*). It is important to note that this observation indicates subcellular proximity but does not constitute direct evidence for a physical interaction.

Functional studies further revealed that RLIM overexpression inhibited MDM2 expression, while RLIM knockdown significantly elevated MDM2 protein levels (Fig. 4*D*). In contrast, MDM2 knockdown did not affect RLIM protein expression (Fig. S4*B*), clarifying that MDM2 regulates RLIM unidirectionally. Moreover, neither overexpression nor knockdown of RLIM significantly altered MDM2 mRNA levels (Fig. 4, *E* and *F*), suggesting that RLIM modulates MDM2 at the post-translational level, likely through its effect on protein stability. To verify this hypothesis, the protein synthesis inhibitor cycloheximide (CHX) was applied to hUCMSCs and 293T cells overexpressing RLIM to monitor changes in MDM2 protein stability (Figs. 4*G* and S4*E*). Western blot analysis demonstrated that RLIM overexpression significantly reduced the half-life of MDM2, while RLIM knockdown increased its steady-state protein levels. Furthermore, treatment with the proteasome inhibitor MG132 partially rescued MDM2 degradation induced by RLIM (Figs. 4*H* and S4*C*), confirming that the degradation is proteasome-dependent. Collectively, these results indicate that RLIM negatively regulates MDM2 stability through the ubiquitin-proteasome system.

The impact of RLIM on MDM2 ubiquitination was assessed in hUCMSCs. The results showed that RLIM knockdown stabilized MDM2 expression and significantly reduced the length of its polyubiquitin chains (Fig. 4*I*). Using protein–protein interaction networks (PPI), this study predicted the binding sites between RLIM and MDM2 (Fig. S4*D*) and identified strong interactions at GLU-271 and LYS-582 (Fig. 4*J*). Further analysis using single amino acid mutants demonstrated that the K582R mutant impaired the RLIM-mediated ubiquitination of MDM2 (Fig. 4*K*), emphasizing the critical role of lysine 582 (K582) in the RLIM-MDM2 interaction. These findings, consistent with cellular experiments, further support RLIM’s role in promoting MDM2 ubiquitination and degradation.

**Figure 5:**
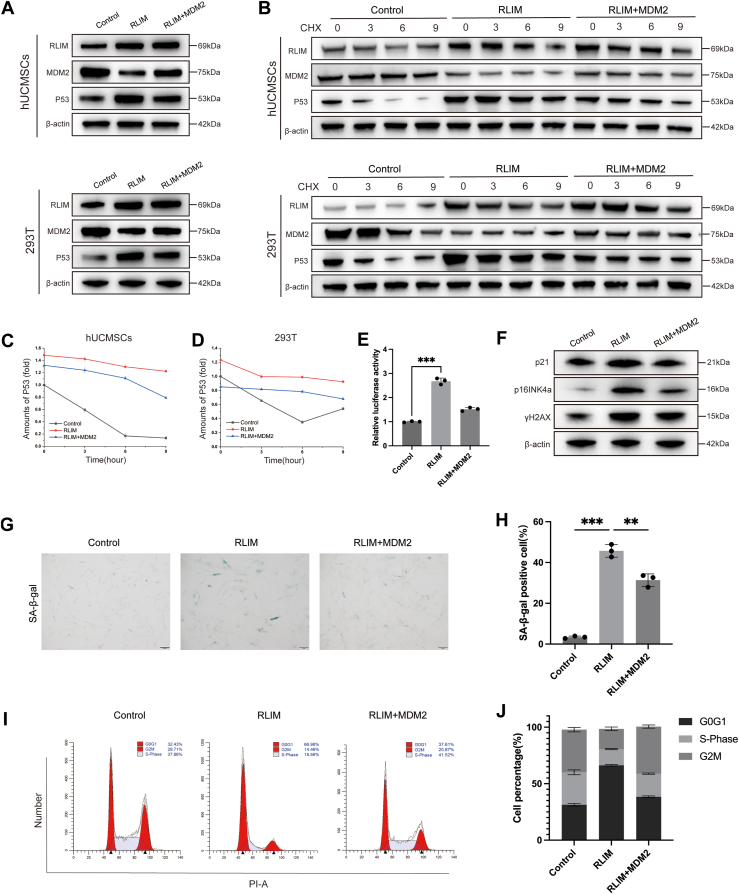
**RLIM regulates p53 stability and activity by promoting MDM2 degradation.***A–D*, CHX chase assays and Western blot analysis showing protein stability and expression levels of MDM2 and p53 in hUCMSCs and 293T cells after RLIM overexpression alone or co-overexpression with MDM2 (n = 3). *E*, dual-luciferase reporter assay measuring p53 transcriptional activity in 293T cells transfected with p53-luc reporter and RLIM expression vector (n = 3). *F*, Western blot analysis of p53 downstream targets and senescence markers (p21, p16^INK4a^, γH2AX) in hUCMSCs after RLIM overexpression or combined RLIM and MDM2 overexpression (n = 3). *G and H*, SA-β-gal staining and quantification of cells overexpressing RLIM alone or co-overexpressing RLIM and MDM2 (n = 3). Scale bar = 100 μm. *I and J*, flow cytometry analysis of cell cycle distribution in cells overexpressing RLIM alone or co-overexpressing RLIM and MDM2 (n = 3). All data are representative of at least three independent experiments and shown as mean ± standard deviation (SD). Statistical analysis was performed using one-way ANOVA followed by Sidak’s multiple comparison test for comparisons among three groups (Control, RLIM^OE^, RLIM^OE^ + MDM2^OE^). For CHX chase assays (*B*–*D*), linear regression analysis was used to calculate MDM2/p53 protein half-lives, and statistical significance of half-life differences among groups was determined by one-way ANOVA followed by Sidak’s multiple comparison test. Significance levels are indicated as ∗*p* < 0.05, ∗∗*p* < 0.01, and ∗∗∗*p* < 0.001.

The p53 tumor suppressor plays a central role in regulating the cell cycle, senescence, and apoptosis, with its activity tightly controlled by the E3 ubiquitin ligase MDM2 (19, 20). MDM2 binds to p53, promoting its ubiquitination and proteasomal degradation, thus maintaining cellular homeostasis under normal conditions (21). Based on this, RLIM may influence p53 protein levels by modulating MDM2 stability under oxidative stress. To test this hypothesis, RLIM was overexpressed alone or in combination with MDM2 in hUCMSCs and 293T cells treated with H_2_O_2_, and the dynamics of MDM2 and p53 were monitored (Figs. 5, *A*–*D* and S4, *F* and *G*). The results showed that RLIM overexpression under H_2_O_2_ treatment significantly shortened the half-life of MDM2, leading to decreased MDM2 protein levels and a concomitant increase in p53 levels. In contrast, co-overexpression of RLIM and MDM2 restored MDM2 stability and, as a consequence, partially reversed the accumulation of p53 seen with RLIM overexpression alone. These results suggest that RLIM indirectly enhances p53 stability by regulating MDM2 degradation.

To further evaluate RLIM’s impact on p53 transcriptional activity, a p53 response element-driven luciferase reporter plasmid (p53-luc) and an RLIM expression vector were co-transfected into 293T cells, followed by luciferase activity measurement using a dual-luciferase reporter assay system (Fig. 5*E*) (22). The data revealed that RLIM overexpression significantly enhanced the luciferase signal of the p53-luc reporter under oxidative stress, indicating that RLIM not only upregulates p53 protein levels but also increases its transcriptional activity. In contrast, co-overexpression of RLIM and MDM2 resulted in a marked reduction in p53-luc activity, suggesting that MDM2 can partially reverse the RLIM-mediated enhancement of p53.

To further investigate the role of the RLIM-MDM2 axis in downstream p53 signaling, RLIM was overexpressed alone or in combination with MDM2 in hUCMSCs and 293T cells, followed by Western blot analysis of p53 target proteins (Fig. 5*F*). The results demonstrated that RLIM overexpression significantly upregulated p53 downstream molecules and senescence-associated markers, such as p21, p16^INK4a^, and γH2AX, a marker of DNA damage associated with cellular senescence (23). However, co-overexpression of RLIM and MDM2 attenuated these upregulations. Additionally, SA-β-gal staining and cell cycle analysis confirmed that RLIM overexpression promoted the senescent phenotype in cells under oxidative stress, while co-expression of MDM2 alleviated this effect (Fig. 5, *G*–*J*).

In summary, under oxidative stress, RLIM overexpression inhibits MDM2 and enhances p53 stability, leading to suppressed autophagy and promoted senescence of hUCMSCs, ultimately impairing stem cell function. Co-expression of MDM2 partially reverses these effects, demonstrating that RLIM’s pro-senescent function depends on the MDM2-p53 pathway.

### RLIM regulates autophagy and senescence of hUCMSCs under oxidative stress *via* MDM2

**Figure 6:**
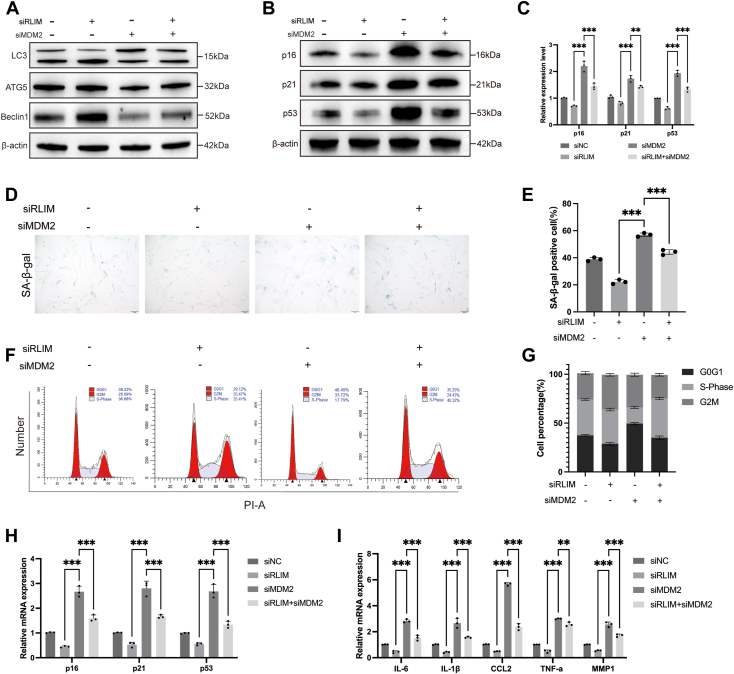
**RLIM knockdown promotes autophagy activation and delays senescence *via* MDM2 in hUCMSCs.***A*, Western blot analysis of autophagy-related proteins ATG5, Beclin1, and LC3-I/II following RLIM knockdown or combined knockdown with MDM2 (n = 3). *B and C*, Western blot analysis of senescence markers p16, p21, and p53 in hUCMSCs following RLIM knockdown or combined knockdown with MDM2 (n = 3). *D and E*, SA-β-gal staining and quantification showing cellular senescence in RLIM knockdown and RLIM/MDM2 co-knockdown groups (n = 3). Scale bar = 100 μm. *F and G*, Flow cytometry analysis of cell cycle distribution assessing senescence-associated arrest in hUCMSCs with RLIM knockdown or RLIM and MDM2 co-knockdown (n = 3). *H and I*, qPCR analysis of senescence-related gene expression levels in hUCMSCs with RLIM knockdown or RLIM and MDM2 co-knockdown (n = 3). All data are representative of at least three independent experiments and shown as mean ± standard deviation (SD). Statistical analysis was performed using one-way ANOVA followed by Sidak’s multiple comparison test for comparisons among four groups (siNC, siRLIM, siMDM2, siRLIM + siMDM2). Significance levels are indicated as ∗*p* < 0.05, ∗∗*p* < 0.01, and ∗∗∗*p* < 0.001.

To explore the molecular mechanisms regulating autophagy under oxidative stress, the interaction between RLIM and MDM2 was investigated. Experimental results demonstrated that RLIM knockdown significantly activated autophagic activity in hUCMSCs under H_2_O_2_-induced oxidative stress, whereas MDM2 inhibition partially reversed this effect (Figs. 6*A* and S5, *A*–*E*). In contrast, RLIM overexpression suppressed autophagy-related protein expression, and co-overexpression of MDM2 alleviated this suppression (Fig. S5, *F*–*I*). These results suggest that RLIM regulates autophagy in hUCMSCs, at least in part, by modulating MDM2 levels under oxidative stress conditions.

Moreover, RLIM knockdown significantly delayed hUCMSCs senescence under oxidative stress, as evidenced by a reduction in SA-β-gal-positive cells, downregulation of senescence markers such as p21, p16^INK4a^, and γH2AX, and restoration of cell cycle progression (Fig. 6, *B*–*I*). However, simultaneous MDM2 knockdown significantly weakened the protective effect of RLIM knockdown against senescence, highlighting the critical role of MDM2 in this process. Conversely, RLIM overexpression accelerated the senescence phenotype in hUCMSCs, an effect that was mitigated by co-overexpression of MDM2 (Fig. S5, *J*–*O*). These results indicate that RLIM regulates both autophagy and senescence through MDM2, playing an essential modulatory role under oxidative stress.

In summary, under oxidative stress, RLIM negatively regulates autophagy in hUCMSCs *via* MDM2, suppressing autophagic activity and promoting senescence, ultimately impairing stem cell function. These findings highlight the pivotal role of the RLIM-MDM2 pathway in regulating stem cell fate under oxidative stress and suggest potential targets for future interventions aimed at preserving stem cell function.

### TRIM36 promotes ubiquitin-dependent degradation of RLIM to regulate the MDM2-p53 axis in hUCMSCs under oxidative stress

To investigate the upstream regulatory mechanism of RLIM under oxidative stress, potential E3 ubiquitin ligases interacting with RLIM were screened (Fig. S6*A*). The results indicated that TRIM36, an E3 ligase containing a RING domain, may harbor binding sites for RLIM. Co-IP assays confirmed the direct interaction between TRIM36 and RLIM, suggesting a critical role for TRIM36 in regulating RLIM protein stability (Figs. S6*B* and S7*A*). In H_2_O_2_-treated hUCMSCs and 293T cells, knockdown or overexpression of TRIM36 led to inverse changes in RLIM protein levels, while RLIM mRNA levels remained largely unchanged (Figs. S6*C*, and S7, *B* and *C*), indicating that this regulation occurs primarily at the post-transcriptional level. Further structure-function analysis revealed that the CC domain (amino acid residues 271–345) of TRIM36 is essential for its binding to RLIM (Fig. S6, *D*–*G*).

Considering TRIM36's biological function as an E3 ligase, and based on the knowledge that its CC domain mediates protein-protein interactions (24, 25, 26), this study examined whether TRIM36 promotes RLIM ubiquitination to regulate its stability (Figs. S6, *H* and *L*, and S7, *D* and *E*). The results demonstrated that TRIM36 enhanced RLIM polyubiquitination in a dose-dependent manner. However, this effect was significantly attenuated upon deletion of the CC domain (ΔCC). Consistently, protein half-life assays revealed that TRIM36 overexpression accelerated RLIM degradation (Figs. S6*I* and S7*F*), supporting a ubiquitin-proteasome–dependent degradation mechanism.

Functional assays indicated that, under oxidative stress, TRIM36 depletion in hUCMSCs significantly upregulated p53 downstream target genes and senescence markers. However, co-knockdown of TRIM36 and RLIM markedly alleviated these effects, suggesting that TRIM36 promotes senescence by modulating the p53 signaling axis *via* RLIM (Fig. S6*J*). Furthermore, TRIM36 deletion shortened the half-life of MDM2 and decreased its protein levels, which was accompanied by increased p53 abundance (Figs. S6, *K* and *M*, and S7, *G* and *H*). Co-knockdown of TRIM36 and RLIM restored the stability of both MDM2 and p53, reversing the effects observed with TRIM36 knockdown alone.

In summary, under oxidative stress, TRIM36 binds to RLIM through its CC domain, promoting its ubiquitin-proteasome-mediated degradation. This process enhances RLIM-mediated autophagy inhibition and senescence promotion, modulating the MDM2-p53 signaling axis and ultimately leading to functional decline in hUCMSCs. This mechanism reveals a novel upstream regulatory network underlying stem cell functional decline under oxidative stress, providing potential targets for intervening aimed at mitigating stem cell senescence.

### RLIM overexpression impairs the wound-healing functions of hUCMSCs under oxidative stress

Wound healing involves the coordinated participation of multiple cell types, including vascular endothelial cells and fibroblasts, which are crucial for tissue regeneration (27). Previous studies, including our own, have demonstrated that stem cell transplantation into wound sites can modulate the function of these cells, promoting angiogenesis and tissue repair (8). To further explore the impact of RLIM on the function of these cell types under oxidative stress, the effects of RLIM on endothelial cells and fibroblasts were assessed (Fig. S8, *A*–*F*). The results revealed that RLIM overexpression in hUCMSCs, when co-cultured with HUVECs under H_2_O_2_ treatment, significantly inhibited endothelial cell tubular structure formation, migration, and scratch wound closure. This indicated that RLIM upregulation impairs the pro-angiogenic properties of hUCMSCs. A similar inhibitory effect was observed in HDF experiments.

Regarding mitochondrial autophagy, RLIM overexpression notably suppressed mitophagic activity in hUCMSCs, leading to the accumulation of damaged mitochondria and reduced mitochondrial function. Consequently, this impairment diminished cell survival and reparative potential under oxidative stress (Fig. S8*G*). In immunomodulatory studies related to diabetic wound healing (Fig. S8, *H* and *I*), RLIM-overexpressing hUCMSCs promoted M1 pro-inflammatory macrophage polarization while reducing the proportion of M2 reparative macrophages, suggesting an adverse effect on the immune microenvironment involved in tissue repair. Flow cytometry analysis further indicated that RLIM overexpression significantly increased the proportion of oxidative stress-induced apoptosis and reduced cell viability (Fig. S8*J*).

In summary, RLIM impairs multiple wound repair-related functions of hUCMSCs, including angiogenesis, cell migration, and immunomodulation, under oxidative stress. This occurs through the inhibition of autophagy and the promotion of senescence. These findings emphasized the critical role of RLIM in dysfunction of hUCMSCs, ultimately contributing to the delayed healing of diabetic wounds.

### RLIM overexpression impairs the wound healing capacity of hUCMSCs under oxidative stress

**Figure 7:**
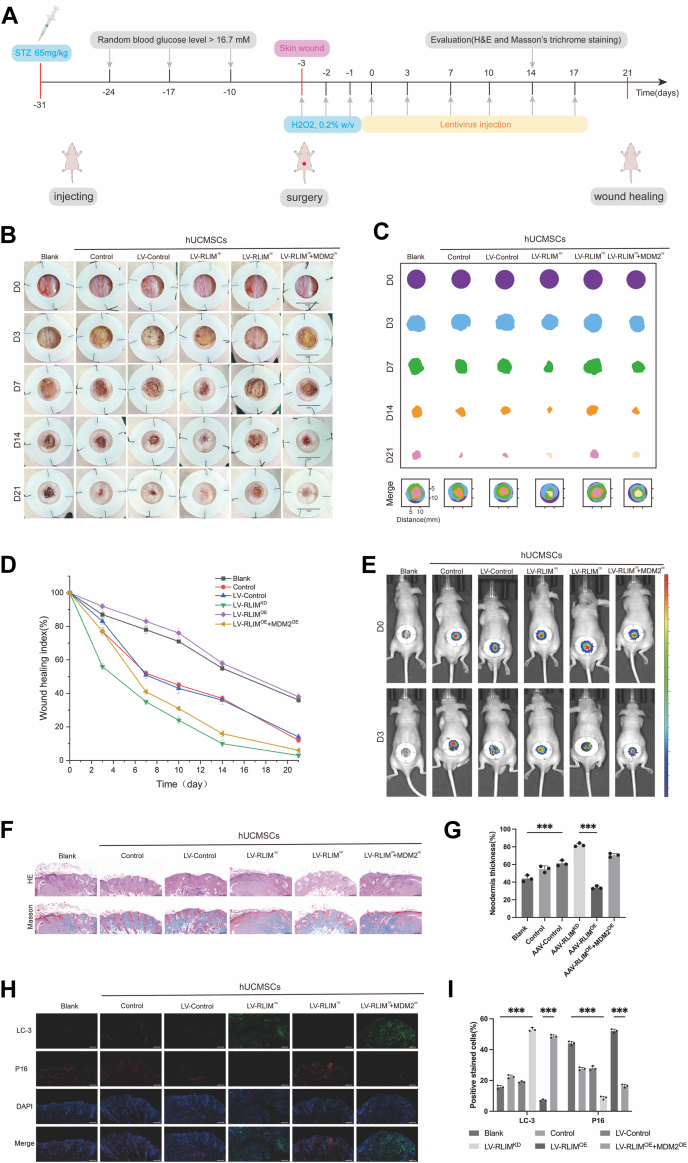
**RLIM downregulation enhances hUCMSC-mediated diabetic wound healing *in vivo*.***A*, schematic diagram of the full-thickness skin defect model and treatment groups. *B*–*D*, representative wound images and quantitative analysis of wound closure rates on days 3, 7, 14, and 21 post-injuries (n = 8 per group). *E*, live-cell tracking assay showing survival of hUCMSCs in diabetic wounds (n = 8 per group). *F and G*, histological analysis of wound tissues by H&E and Masson’s trichrome staining on day 14 post-surgery. Scale bar = 50 μm. *H and I*, Immunofluorescence staining of the autophagy marker LC3 and the senescence marker p16 in wound tissues. Scale bar = 50 μm. All data are representative of at least three independent experiments (for in vitro-derived hUCMSCs) or biological replicates (for *in vivo* animals) and shown as mean ± standard deviation (SD). Statistical analysis was performed using one-way ANOVA followed by Sidak’s multiple comparison test for comparisons among multiple treatment groups. For time-dependent wound closure rate (*D*), two-way repeated-measures ANOVA followed by Sidak’s multiple comparison test was used to analyze differences among groups across different time points (3, 7, 14, 21 days). Significance levels are indicated as ∗*p* < 0.05, ∗∗*p* < 0.01, and ∗∗∗*p* < 0.001.

Wound healing is severely impaired under diabetic and oxidative stress conditions, primarily due to compromised stem cell function and reduced regenerative capacity. While hUCMSCs have been shown to promote wound repair, the role of RLIM in modulating their therapeutic effects remains unclear. To address this, full-thickness skin defect models were established on the backs of diabetic nude mice, followed by subcutaneous injections of LV-RLIM^KD^ (RLIM knockdown), LV-RLIM^OE^ (RLIM overexpression), LV-RLIM^OE^ + MDM2^OE^ (RLIM and MDM2 co-overexpression), or an equal volume of PBS as control (Fig. 7*A*). The results revealed that under H_2_O_2_-induced oxidative stress, the wound closure rate in the RLIM overexpression group was significantly delayed, and wound healing was impaired compared to the untreated hUCMSCs group (Fig. 7, *B*–*D*). Additionally, live-cell tracking assays demonstrated that RLIM-overexpressing hUCMSCs exhibited markedly reduced survival in the wound area (Fig. 7*E*). Histological analysis conducted on the 14th day after the operation further confirmed that the RLIM overexpression group displayed limited skin tissue regeneration, reduced collagen deposition, and decreased vascular density (Figs. 7, *F* and *G*, and S9, *A* and *B*). Regarding immunomodulation, RLIM overexpression resulted in a high proportion of M1 macrophages and a reduced proportion of M2 macrophages. This shift was accompanied by decreased secretion of pro-reparative factors, such as IL-10, VEGF, and TGF-β, along with increased levels of pro-inflammatory cytokines, including TNF-α, IL-1β, and IL-6 (Fig. S9, *C*–*E*). Moreover, RLIM-overexpressing hUCMSCs exhibited enhanced senescence phenotypes and suppressed autophagy activity (Fig. 7, *H* and *F*). In summary, these experiments demonstrate that under oxidative stress, RLIM impairs the wound healing potential of hUCMSCs by promoting cellular senescence, inhibiting autophagy, and reducing cell viability and angiogenesis, thereby exacerbating diabetic wound repair deficits.

## Discussion

Chronic diabetic wounds are characterized by impaired healing, primarily due to stem cell dysfunction and premature senescence. Previous studies have demonstrated that hUCMSCs can promote tissue repair and angiogenesis (28); however, the diabetic and oxidative stress microenvironment severely compromises their survival, differentiation, and regenerative potential. Several reports indicate that autophagy acts as a protective mechanism, enabling hUCMSCs to resist apoptosis and preserve functionality (29, 30). However, the upstream regulators linking ubiquitin-mediated degradation to autophagy and senescence remain poorly understood, and the precise molecular mechanisms governing stem cell adaptation to oxidative stress are largely unexplored.

RLIM, a classical E3 ubiquitin ligase involved in transcriptional regulation and various diseases, including cancer, has not been studied in the context of stem cell senescence and autophagy under oxidative stress. In this study, RLIM serves as a key modulator in hUCMSCs by promoting MDM2 ubiquitination and degradation, leading to p53 stabilization, suppression of autophagy, and accelerated cellular senescence. This study addresses a critical gap in understanding how ubiquitin signaling regulates stress-induced stem cell aging, a mechanism not previously explored in MSC research.

TRIM36 was identified as an upstream E3 ligase that interacts with the CC domain of RLIM to mediate its polyubiquitination and degradation. While previous studies have focused on single E3 ligase-mediated regulation of downstream targets, the hierarchical E3-E3 network revealed in this study offers a novel perspective on multi-layered ubiquitin regulation in stem cells. This dual regulation provides precise control over autophagy and senescence in oxidative stress conditions, expanding the conceptual framework beyond previous studies.

Functionally, RLIM overexpression impairs hUCMSCs-mediated angiogenesis, migration, and immunomodulatory capacity *in vitro* as well as delays wound healing in diabetic models. While previous MSC transplantation studies have demonstrated beneficial effects on wound repair, they did not examine how oxidative stress-induced ubiquitin signaling impacts stem cell function *in vivo*. This study directly addressed this gap, providing mechanistic insights with translational implications.

Despite these advancements, several limitations persist. This study primarily focused on RLIM in regulating autophagy and senescence. However, other E3 ligases or signaling pathways likely contribute to stem cell dysfunction under oxidative stress, warranting further investigation. Additionally, the *in vivo* studies were limited to diabetic wound models, and the applicability of this regulatory axis to other tissue types or metabolic disorders remains to be tested. Furthermore, although mechanistic insights are provided, the pharmacological modulation of RLIM has not yet been evaluated, limiting immediate clinical translation.

In conclusion, this study integrated ubiquitination, autophagy, and senescence pathways, systematically elucidating the RLIM-MDM2-p53 axis as a key regulator of hUCMSCs function under oxidative stress. The findings clarify previously unaddressed mechanisms, particularly how E3 ligase regulation orchestrates autophagy and senescence, and propose actionable targets to enhance stem cell therapies for diabetic chronic wounds. Future research should focus on exploring additional regulators within this network, validating these findings in diverse disease models, and developing pharmacological strategies to fine-tune ubiquitin signaling for clinical translation, ultimately improving the efficacy of MSC-based regenerative therapies.

## Experimental procedures

### Cell culture and treatment

Human umbilical cord stem cells (hUCMSCs) were obtained from Shandong Qilu Cell Therapy Engineering Technology Co., Ltd. These cells were cultured in StemGro Mesenchymal Stem Cell Medium (BasaMedia) supplemented with fetal bovine serum, in a humidified incubator at 37 °C with 5% CO_2_. Based on previous studies, a concentration of 200 μM H_2_O_2_ was used *in vitro* to simulate an oxidative stress environment.

### Autophagic flux assay

hUCMSCs were transduced with a tandem fluorescent RFP-GFP-LC3B adenovirus and subsequently counterstained with DAPI for nucleus visualization. The number of green fluorescent protein (GFP) and monomeric red fluorescent protein (RFP) puncta were manually counted to assess autophagic flux.

### Western blot analysis

For protein analysis, cell lysates were separated by 4 to 12% gradient SDS-PAGE gels and transferred to nitrocellulose membranes. The membranes were incubated overnight at 4 °C with primary antibodies diluted 1:2000, followed by incubation with secondary antibodies diluted 1:4000 at room temperature for 1.5 h. Protein bands were detected using Tanon scanning equipment (Tanon Science & Technology). Detailed information about the antibodies is provided in Table S1.

### LC3 immunofluorescence staining

For IF staining, hUCMSCs were seeded in 24-well plates and cultured according to the experimental design. After treatment, cells were washed three times with phosphate-buffered saline (PBS) and fixed with 4% paraformaldehyde at room temperature for 10 min. Cells were then permeabilized with PBS containing 0.5% Triton X-100 for 10 min and blocked with a rapid blocking solution for 20 min at room temperature. The cells were incubated overnight at 4 °C with a primary antibody against LC3 (dilution 1:200). The following day, cells were washed three times with PBS and incubated with the corresponding fluorescent secondary antibody (dilution 1:200) for 1 h at room temperature in the dark (31). Nuclei were stained with DAPI for 5 min. Fluorescence images were captured using a microscope (Olympus, Center Valley, USA).

### qRT-PCR

Total RNA was extracted from hUCMSCs using TRIzol reagent (VR603, VicMed). RNA was reverse-transcribed into cDNA using a reverse transcription kit (HY-K0501, MCE). Quantitative PCR was performed on a LightCycler 480 instrument (Roche) using a premixed SYBR Green Master Mix (VFP101, VicMed). qRT-PCR was used to assess mRNA expression levels. Each reaction was repeated at least three times independently. Data were analyzed using the 2^−ΔΔCT^ method. Primers for RT-qPCR were designed using the online tool Primer3web, as listed in Table S2.

### Senescence-associated β-galactosidase (SA-β-gal) staining

The SA-β-gal staining kit (Beyotime) was used according to the manufacturer’s instructions. hUCMSCs seeded in six-well plates were incubated with 1 ml of SA-β-gal staining solution overnight at 37 °C in a CO_2_-free incubator. The following day, cells were washed three times with PBS and observed under a microscope. SA-β-gal signals were quantified using ImageJ software.

### Cell cycle flow cytometry

The Cell Cycle Assay Kit (APE x BIO) was used as per the manufacturer's guidelines. hUCMSCs were collected, washed once with pre-chilled PBS, and then fixed overnight at 4 °C with 70% ethanol. After fixation, cells were washed with PBS and incubated with 0.5 ml staining solution containing Staining Buffer, PI, and RNase A at 37 °C for 30 min in the dark. Cell cycle distribution was analyzed using a Canto II flow cytometer (BD), with data processed using ModFit LT 5.0 software. Each experiment was conducted at least three times.

### siRNA transfection

hUCMSCs were seeded in six-well plates and cultured in serum-containing specialized medium at 37 °C in a 5% CO_2_ incubator. When cell confluence reached 60%-70%, transient transfection was performed using lipofectamine_2000 (Invitrogen) following the manufacturer's instructions. siRNAs and control siRNA were purchased from GenePharma, with sequences provided in Table S3. For each well, 50 nM siRNA was incubated with lipofectamine_2000 in serum-free specialized medium at room temperature for 20 min to form complexes, which were then added to the cells and incubated at 37 °C for 8 h. After transfection, the medium was replaced with serum-containing specialized medium for an additional 24 to 48 h to achieve siRNA-mediated gene silencing. Gene silencing efficiency was assessed by qRT-PCR 48 h post-transfection.

### Molecular docking analysis

To predict the interaction interface between RLIM and MDM2, protein-protein molecular docking was performed. The docking procedure was carried out using the HDOCK online server, whose docking algorithm integrates known structural information from the Protein Data Bank (PDB) with *ab initio* free docking strategies. The sequences of RLIM and MDM2 were submitted to the server for a global search. The results were ranked according to the HDOCK scoring function, which combines terms for binding energy, desolvation effect, and electrostatics. The final complex model was selected from the top-ranking candidates based on the best docking score and the plausibility of the interactions. This model revealed critical interactions between GLU-271 (of RLIM) and LYS-582 (of MDM2). The final complex structure presented in Figure 4*J* was visualized and analyzed using the PyMOL molecular graphics system.

### Co-immunoprecipitation and mass spectrometry

The Co-immunoprecipitation (Co-IP) and mass spectrometry (MS) analysis was conducted by Meiji Biopharmaceutical Technology Co., Ltd. Protein complexes were immunoprecipitated from cell lysates using specific antibodies, then separated by SDS-PAGE. Specific protein bands were excised, subjected to in-gel tryptic digestion, and analyzed by mass spectrometry. The resulting peptide fragments were identified and analyzed using proprietary methods, with peptide identification confidence indicated by the "MS score," reflecting the reliability of peptide matching.

### Co-culture of cells

hUCMSCs were cultured in mesenchymal stem cell medium with fetal bovine serum and incubated at 37 °C in a humidified 5% CO_2_ incubator for 24 h. During this time, conditioned medium (supernatant) from hUCMSCs was collected and added to macrophages, fibroblasts, and endothelial cells for indirect co-culture experiments.

### Tube formation assay

After thawing Matrigel (BD Biosciences) on ice, 200 μl was added to each well of a 48-well plate and incubated at 37 °C for 30 min to allow gelation. Subsequently, 3 × 10^5^ pretreated HUVECs were seeded onto the solidified Matrigel and cultured in a 37 °C incubator with 5% CO_2_ for 4 to 6 h. Tube formation was observed and imaged using an inverted fluorescence microscope (Olympus). The total tube length, number of junctions, and percentage of tube area were quantified using ImageJ software.

### ELISA

Levels of IL-10, VEGF, TGF-β, TNF-α, IL-1β, and IL-6 were measured using ELISA. Skin biopsies were collected starting from day 7 and processed according to the manufacturer’s instructions for each ELISA kit (Abcam). The mice used in this study were obtained from the Animal Center of Xuzhou Medical University (Xuzhou). Optical density (OD) values for each well were measured using an AMR-100 automatic microplate reader (Hangzhou Aosheng Instrument).

### Establishment of a diabetic wound model in nude mice

Six-week-old male nude mice were purchased from Jiangsu Jicui Yaokang Biotechnology Co., Ltd and housed under sterile conditions. All animal experiments were approved by the Animal Care and Ethics Committee of Xuzhou Medical University (project number 202505T017). After 12 h of fasting, mice were intraperitoneally injected with streptozotocin (STZ, 65 mg/kg; Sigma) dissolved in a pH 4.5 citrate buffer to induce diabetes. Blood glucose levels were monitored weekly, and mice with blood glucose consistently exceeding 16.7 mmol/L for at least 4 weeks were considered diabetic models.

Following anesthesia, a full-thickness skin wound with a diameter of 1.0 cm was created on the backs of the nude mice. To mimic oxidative stress conditions, local oxidative stress was induced at the wound site by topical application of hydrogen peroxide (H_2_O_2_, 0.2% w/v) immediately after wounding and once daily for three consecutive days.

### Assessment of wound closure

Forty-eight diabetic nude mice were divided into six groups, with eight mice per group. The groups included: Blank group (diabetic wound healing model), hUCMSCs treatment control group (diabetic mice injected with untreated hUCMSCs), LV-Control group (injected with LV-Control), LV-RLIMKD group (injected with RLIM-knockdown hUCMSCs), LV-RLIMOE group (injected with RLIM-overexpressing hUCMSCs), and LV-RLIMOE + MDM2OE group (injected with hUCMSCs overexpressing both RLIM and MDM2). Wound images were captured on days 0, 3, 7, 14, and 21 post-surgery and analyzed using ImageJ software. For data measurement, three mice were randomly selected per group. The wound closure rate was calculated as: wound closure index (%) = (1 − unhealed wound area/original wound area) × 100%.

### H&E and Masson staining

Mouse skin tissues were collected and immediately fixed in 4% paraformaldehyde for 24 h. After fixation, the tissues were embedded in paraffin and sectioned at a thickness of 3 μm. The paraffin sections were deparaffinized, immersed in water, and stained with hematoxylin for 3 to 5 min, followed by rinsing. The sections were then dehydrated in 85% and 95% ethanol for 5 min each, stained with eosin for 5 min, and subjected to Masson’s trichrome staining. After rinsing, the sections were observed under a microscope (32).

### Statistical analysis

Statistical analyses were performed using OriginPro 2024 and GraphPad Prism 9. Data are presented as mean ± standard deviation (SD). Statistical differences were assessed using Student's *t* test or one-way analysis of variance (ANOVA). *p* < 0.05 was considered statistically significant.

## Ethics

The animal studies were carried out at the Experimental Animal Center of Xuzhou Medical University (Project No.202505T017).

## Data availability

All data are contained within the manuscript.

## Supporting information

This article contains supporting information.

## Conflicts of interest

The authors declare that they have no conflicts of interest with the contents of this article.
