# Supplementary material for: The RING E3 ligase RLIM drives oxidative stress-induced stem cell dysfunction through MDM2-p53 signaling
Source: J Biol Chem. 2026 Mar 20;302(5):111391. doi: 10.1016/j.jbc.2026.111391 (PMC13122216; doi:10.1016/j.jbc.2026.111391)
Supplement: Supporting Information [file mmc1.docx]

**Supporting Information**

**The RING E3 Ligase RLIM Drives Oxidative Stress-Induced Stem Cell Dysfunction through MDM2-p53 Signaling**

*Xiaoyue Li^1,2,5^, Caiqi Shen^1,2,5^, Yajie Li^2,3^, Lijun Wu^2,3^, Jia Gao^1,2^, Dong Zhu^4^, Dong Dong^3^, Feifei Chen^3,*^, Peisheng Jin^1,*^*

^1^ Department of Plastic Surgery, Affiliated Hospital of Xuzhou Medical University, Xuzhou, China

^2^ Xuzhou Medical University, Xuzhou, China

^3^ Jiangsu Center for the Collaboration and Innovation of Cancer Biotherapy, Cancer Institute, Xuzhou Medical University, Xuzhou, Jiangsu, China

^4^ Center for Cleft Lip and Palate Treatment, Plastic Surgery Hospital, Chinese Academy of Medical Sciences and Peking Union Medical College

^5^ These authors contributed equally this article

^*^ Corresponding author: Peisheng Jin, [peishengjin@xzhmu.edu.cn](mailto:peishengjin@xzhmu.edu.cn); Feifei Chen, chenfeifei@xzhmu.edu.cn

**Supplementary Figure 1**

 **Supplementary Figure S1. Autophagy analysis in hUCMSCs under oxidative stress.** (**A, B**) Western blot analysis and quantification of autophagy-related proteins (LC3, ATG5, Beclin1) in hUCMSCs treated with different concentrations of H₂O₂ (0, 100, 200, 300 μM) (n = 3). (**C**) Immunofluorescence staining of LC3 in hUCMSCs exposed to various H_2_O_2_ concentrations (0, 100, 200, 300 μM) (n = 3). Scale bar = 50μm. (**D**) Assessment of autophagic flux in hUCMSCs exposed to different H_2_O_2_ concentrations (0, 100, 200, 300 μM) (n = 3). Scale bar = 5μm. (**E, F**) Transmission electron microscopy images showing autophagosomes in hUCMSCs treated with varying concentrations of H₂O₂ (0, 100, 200, 300 μM) (n = 3). (**G**) Validation of RLIM knockdown efficiency by qPCR. (**H**) Validation of RLIM knockdown efficiency by Western blot (n = 3). All data are representative of at least three independent experiments and shown as mean ± standard deviation (SD). Statistical analysis was performed using one-way ANOVA followed by Sidak’s multiple comparison test for comparisons among multiple H₂O₂ concentration groups (0, 100, 200, 300 μM) and RLIM knockdown groups (siNC, siRLIM#1, siRLIM#2, siRLIM#3). Significance levels are indicated as *p < 0.05, **p < 0.01, and ***p < 0.001.

**Supplementary Figure 2**

**Supplementary Figure S2.** **RLIM overexpression promotes cellular senescence under oxidative stress.** (**A**) GSEA enrichment analysis of RNA-seq data showing significant enrichment of p53 and senescence-related pathways in oxidative stress-treated hUCMSCs. (**B, C**) Western blot analysis and quantification of senescence-related proteins (p16, p21, and p53) in RLIM-overexpressing hUCMSCs (n = 3). (**D, E**) Representative images and quantification of SA-β-gal staining showing the proportion of senescent cells in RLIM-overexpressing hUCMSCs (n = 3). Scale bar = 100μm. (**F, G**) Cell cycle analysis of RLIM-overexpressing hUCMSCs by flow cytometry (n = 3). (**H, I**) qPCR analysis of senescence-related gene expression levels in RLIM-overexpressing hUCMSCs (n = 3). All data are representative of at least three independent experiments and shown as mean ± standard deviation (SD). Statistical analysis was performed using Student’s t-test for pairwise comparisons between Vector and RLIM^OE^ groups. Significance levels are indicated as *p < 0.05, **p < 0.01, and ***p < 0.001.

**Supplementary Figure 3**

 **Supplementary Figure S3. RLIM regulates autophagic activity under oxidative stress.** (**A**) Immunofluorescence staining of LC3 in hUCMSCs after RLIM knockdown (n = 3). Scale bar = 50μm. (**B**) Quantification of mature autophagolysosome proportion after RLIM knockdown (n = 3). Scale bar = 5μm. (**C, D**) Western blot detection of ATG5, Beclin1, and LC3-I/II protein levels following RLIM knockdown (n = 3). (**E, F**) Transmission electron microscopy images showing enhanced autophagolysosome formation following RLIM knockdown (n = 3). (**G**) Immunofluorescence staining of LC3 in hUCMSCs after RLIM overexpression (n = 3). Scale bar = 50μm. (**H**) Quantification of mature autophagolysosome proportion after RLIM overexpression (n = 3). Scale bar = 5μm. (**I, J**) Western blot detection of ATG5, Beclin1, and LC3-I/II protein levels following RLIM overexpression (n = 3). (**K, L**) Transmission electron microscopy images showing autophagolysosome formation following RLIM overexpression (n = 3). All data are representative of at least three independent experiments and shown as mean ± standard deviation (SD). Statistical analysis was performed using Student’s t-test for pairwise comparisons between siNC and siRLIM groups, or between Vector and RLIM^OE^ groups. Significance levels are indicated as *p < 0.05, **p < 0.01, and ***p < 0.001.

**Supplementary Figure 4**

 **Supplementary Figure S4. RLIM mediates ubiquitin-dependent degradation of MDM2.** (**A**) Co-IP confirming the interaction between RLIM and MDM2 in 293T cells (n = 3). (**B**) Western blot analysis showing that MDM2 knockdown does not affect RLIM protein levels (n = 3). (**C**) Western blot showing MG132-mediated restoration of MDM2 protein levels in RLIM-overexpressing 293T cells (n = 3). (**D**) Protein-protein interaction (PPI) network analysis predicting the interaction between RLIM and MDM2. (**E**) CHX chase assay demonstrating the effect of RLIM overexpression or knockdown on the half-life of MDM2 protein in 293T cells (n = 3). (**F-G**) The densitometric quantification of RLIM, MDM2, and p53 protein expression in hUCMSCs (**F**) and 293T cells (**G**) under conditions of RLIM overexpression with or without MDM2 co-expression and oxidative stress treatment, as corresponding to Figure 5A-D (n = 3). (**H**) Quantitative presentation of the p21, p16INK4a and γH2AX protein signals as corresponding to Figure 5F (n = 3). All data are representative of at least three independent experiments and shown as mean ± standard deviation (SD). Statistical analysis was performed using Student’s t-test for pairwise comparisons (Vector vs RLIM^OE^, siNC vs siRLIM, RLIM^OE^ vs RLIM^OE^ + MG132) and one-way ANOVA followed by Sidak’s multiple comparison test for comparisons among Control, RLIM, and RLIM + MDM2 groups. Significance levels are indicated as *p < 0.05, **p < 0.01, ***p < 0.001, and “ns” indicates no significant difference (p ≥ 0.05).

**Supplementary Figure 5**

 **Supplementary Figure S5. RLIM modulates autophagy and cellular senescence *via* MDM2 in hUCMSCs.** (**A**) Western blot analysis of autophagy-related proteins (n = 3). (**B**) Immunofluorescence staining of LC3 showing autophagic activity in hUCMSCs after RLIM knockdown or combined RLIM and MDM2 knockdown (n = 3). Scale bar = 50μm. (**C**) Confocal microscopy images monitoring autophagosome dynamics under RLIM knockdown and co-knockdown with MDM2 (n = 3). Scale bar = 5μm. (**D, E**) Transmission electron microscopy images of autolysosomes in cells with RLIM knockdown or RLIM and MDM2 co-knockdown (n = 3). (**F**) Immunofluorescence staining of LC3 demonstrating reduced autophagic activity in hUCMSCs after RLIM overexpression or combined RLIM and MDM2 overexpression (n = 3). Scale bar = 50μm. (**G, I**) Western blot analysis of autophagy-related proteins ATG5, Beclin1, and LC3-I/II following RLIM overexpression or combined RLIM and MDM2 overexpression (n = 3). (**H**) Confocal microscopy images monitoring autophagosome dynamics under RLIM overexpression and co-overexpression with MDM2 (n = 3). Scale bar = 5μm. (**J, K**) Western blot analysis of senescence markers p16, p21, and p53 in hUCMSCs following RLIM overexpression or combined overexpression with MDM2 (n = 3). (**L, N**) SA-β-gal staining and quantification showing cellular senescence in RLIM overexpression and RLIM/MDM2 co-overexpression groups (n = 3). Scale bar = 100μm. (**M, O**) Flow cytometry analysis of cell cycle distribution assessing senescence-associated arrest in hUCMSCs with RLIM overexpression or RLIM and MDM2 co-overexpression (n = 3). All data are representative of at least three independent experiments and shown as mean ± standard deviation (SD). Statistical analysis was performed using one-way ANOVA followed by Sidak’s multiple comparison test for comparisons among siNC, siRLIM, siMDM2, siRLIM + siMDM2 groups and Control, RLIM^OE^, MDM2^OE^, RLIM^OE^ + MDM2^OE^ groups. Significance levels are indicated as *p < 0.05, **p < 0.01, and ***p < 0.001.

**Supplementary Figure 6**

**Supplementary Figure S6. TRIM36 promotes ubiquitination and degradation of RLIM, regulating the MDM2-p53 axis.** (**A**) Immunoprecipitation (IP) combined with mass spectrometry (MS) identifying E3 ubiquitin ligases interacting with RLIM in hUCMSCs. (**B**) Co-immunoprecipitation (Co-IP) confirming the direct interaction between TRIM36 and RLIM in hUCMSCs (n = 3). (**C**) Western blot analysis showing inverse correlation between TRIM36 and RLIM protein levels following TRIM36 knockdown or overexpression (n = 3). (**D**) Schematic overview of TRIM36 domains and deletion mutants. (**E**) Schematic overview of RLIM domains and deletion mutants. (**F**) Western blot detecting interaction between Flag-TRIM36 and HA-RLIM using full-length and deletion mutants (n = 3). (**G**) Western blot detecting interaction between Flag-RLIM and HA-TRIM36 using full-length and deletion mutants (n = 3). (**H**) TRIM36 promotes polyubiquitination of RLIM in a dose-dependent manner in hUCMSCs (n = 3). (**I**) CHX chase assay showing accelerated degradation of RLIM upon TRIM36 overexpression in hUCMSCs (n = 3). (**J**) Western blot analysis of p53 downstream targets and senescence markers after TRIM36 knockdown alone or combined knockdown with RLIM in hUCMSCs (n = 3). (**K**) Western blot analysis showing increased p53 protein levels after TRIM36 knockdown, reversed by co-knockdown of TRIM36 and RLIM in hUCMSCs (n = 3). (**L**) Ubiquitination assay demonstrating that deletion of the CC domain (ΔCC) of TRIM36 significantly reduces its ability to promote RLIM polyubiquitination in hUCMSCs (n = 3). (**M**) CHX chase assay showing decreased MDM2 protein stability after TRIM36 knockdown, which is rescued by co-knockdown of TRIM36 and RLIM in hUCMSCs (n = 3). All data are representative of at least three independent experiments and shown as mean ± standard deviation (SD). Statistical analysis was performed using Student’s t-test for pairwise comparisons (siNC vs siTRIM36, Vector vs TRIM36^OE^) and one-way ANOVA followed by Sidak’s multiple comparison test for comparisons among siNC, siTRIM36, siTRIM36 + siRLIM groups and full-length vs deletion mutant groups. Significance levels are indicated as *p < 0.05, **p < 0.01, and ***p < 0.001.

**Supplementary Figure 7**

**Supplementary Figure S7. TRIM36 promotes ubiquitin-dependent degradation of RLIM to regulate the MDM2-p53 axis.** (**A**) Co-IP confirming the interaction between TRIM36 and RLIM in 293T cells (n = 3). (**B, C**) qRT-PCR analysis showing that TRIM36 knockdown or overexpression does not affect RLIM mRNA levels (n = 3). (**D**) Ubiquitination assay in 293T cells showing dose-dependent promotion of RLIM polyubiquitination by TRIM36 (n = 3). (**E**) Ubiquitination assay demonstrating that deletion of the CC domain (ΔCC) of TRIM36 significantly reduces its ability to promote RLIM polyubiquitination in 293T cells (n = 3). (**F**) CHX chase assay validating accelerated degradation of RLIM upon TRIM36 overexpression in 293T cells (n = 3). (**G, H**) CHX chase assay and Western blot analysis showing that TRIM36 knockdown decreases MDM2 stability and increases p53 expression, which is reversed by co-knockdown of RLIM in 293T cells (n = 3). All data are representative of at least three independent experiments and shown as mean ± standard deviation (SD). Statistical analysis was performed using Student’s t-test for pairwise comparisons (siNC vs siTRIM36, Vector vs TRIM36^OE^) and one-way ANOVA followed by Sidak’s multiple comparison test for comparisons among siNC, siTRIM36, siTRIM36 + siRLIM groups. Significance levels are indicated as *p < 0.05, **p < 0.01, and ***p < 0.001.

**Supplementary Figure 8**

 **Supplementary Figure S8.** **RLIM-overexpressing hUCMSCs impair wound repair through multiple cellular mechanisms.** (**A-F**) Functional analyses showing that under oxidative stress, co-culture of RLIM-overexpressing hUCMSCs with HUVECs or fibroblasts inhibits endothelial tube formation, cell migration, and scratch wound healing (n = 3). Scale bar = 100μm. (**G**) Analysis of mitochondrial autophagy in RLIM-overexpressing hUCMSCs (n = 3). Scale bar = 5μm. (**H, I**) Flow cytometry analysis of the immunomodulatory effects of RLIM-overexpressing hUCMSCs on macrophage polarization (n = 3). (**J**) Flow cytometry analysis of apoptosis in cells treated with RLIM-overexpressing hUCMSCs under oxidative stress conditions (n = 3). All data are representative of at least three independent experiments and shown as mean ± standard deviation (SD). Statistical analysis was performed using Student’s t-test for pairwise comparisons between Vector and RLIM^OE^ groups. For time-dependent scratch closure rate (B, E), two-way repeated-measures ANOVA followed by Sidak’s multiple comparison test was used to analyze differences between groups across time points. Significance levels are indicated as *p < 0.05, **p < 0.01, and ***p < 0.001.

**Supplementary Figure 9**

**Supplementary Figure S9. Immunomodulatory, proliferative, and angiogenic effects of RLIM-regulated hUCMSCs in diabetic wound healing.** (**A, B**) Immunofluorescence staining for iNOS and CD206 demonstrating macrophage polarization in wound tissues. Scale bar = 50μm. (**C, D**) Immunohistochemical analysis of Ki67 and CD31 showing cell proliferation and angiogenesis in wound tissues. Scale bar = 50μm. (**E**) ELISA analysis of pro-repair cytokines (IL-10, VEGF, TGF-β) and pro-inflammatory cytokines (TNF-α, IL-1β, IL-6) in the wound microenvironment (n = 3). All data are representative of biological replicates (n = 8 per group) and shown as mean ± standard deviation (SD). Statistical analysis was performed using one-way ANOVA followed by Sidak’s multiple comparison test for comparisons. Significance levels are indicated as *p < 0.05, **p < 0.01, and ***p < 0.001.

**Supplementary Figure 10: unedited blot images of each figure**

**Supplementary Table 1. Antibodies Used in this Study**

| Antibodies | SOURCE | IDENTIFIER |
| --- | --- | --- |
| Anti-p16  Anti-p21  Anti-p53  Anti-LC3 | Abcam  Proteintech  Proteintech  Proteintech | Cat#ab51243  Cat#10355-1-AP  Cat#10442-1-AP  Cat#14600-1-AP |
| Anti-ATG5  Anti-Beclin-1  Anti-β-actin  Anti-RLIM  Anti-MDM2  Anti-Ubiquitin  Anti-p16INK4a  Anti-γH2AX  Anti-TRIM36  Anti-Ki-67  Anti-CD31  Anti-iNOS  Anti-CD206  Anti-Flag  Anti-HA  Anti-His | Proteintech  Proteintech  Proteintech  Proteintech  Proteintech  Proteintech  Proteintech  Proteintech  Proteintech  Proteintech  Proteintech  Proteintech  Proteintech  Proteintech  Proteintech  Biodragon | Cat#10181-1-AP  Cat#11306-1-AP  Cat#20536-1-AP  Cat#16121-1-AP  Cat#27883-1-AP  Cat#10201-2-AP  Cat#10883-1-AP  Cat#83307-2-RR  Cat#25913-1-AP  Cat#27309-1-AP  Cat#11265-1-AP  Cat#22226-1-AP  Cat#18704-1-AP  Cat#66008-4-Ig  Cat#51064-2-AP  Cat#B1004 |

**Supplementary Table 2. Primers Sequence**

| Gene Name | Sequence |
| --- | --- |
| ACTB | 5´-GATGCGTTGTTACAGGAAGTCC-3´ (forward)  5´-GGCACGAAGGCTCATCATTCA-3´ (reverse) |
| IL-6  TNF-α  IL-1β  CCL2  MMP1  P16  P21  P53  IL-10  VEGF  TGF-β  CDKMA  MDM2  RLIM  KLF4  ATF3 | 5´-TGAGGAGACTTGCCTGGTGAA-3´ (forward)  5´-CAGCTCTGGCTTGTTCCTCAC-3´ (reverse)  5´-TATCCTGGGGGACCCAATGT-3´ (forward)  5´-AAAAGAAGGCACAGAGGCCA-3´ (reverse)  5´-CTGTACCTGTCCTGCGTGTT-3´(forward)  5´-GGGAACTGGGCAGACTCAAA-3´(reverse)  5´-AGCAGCAAGTGTCCCAAAGA-3´(forward)  5´-GGTGTCTGGGGAAAGCTAGG-3´(reverse)  5´-ATCGGGGCTTTGATGTACCC-3´(forward)  5´-CGATGGGCTGGACAGGATTT-3´(reverse)  5´-CACTCTCACCCGACCCGTG-3´(forward)  5´-ATCTATGCGGGCATGGTTACTG-3´(reverse)  5´-CTCAAATCGTCCAGCGACCT-3´(forward)  5´-GACTCCTTGTTCCGCTGCTA-3´(reverse)  5´-CGCTTCGAGATGTTCCGAGA-3´(forward)  5´-CTTCAGGTGGCTGGAGTGAG-3´(reverse)  5´-GACTTTAAGGGTTACCTGGGTTG-3´(forward)  5´-TCACATGCGCCTTGATGTCTG-3´(reverse)  5´-ACAACAAATGTGAATGCAGACCA-3´(forward)  5´-GAGGCTCCAGGGCATTAGAC-3´(reverse)  5´-TGATGTCACCGGAGTTGTGC-3´(forward)  5´-CGGTAGTGAACCCGTTGATG-3´(reverse)  5´-CTCAAATCGTCCAGCGACCT-3´(forward)  5´-GACTCCTTGTTCCGCTGCTA-3´(reverse)  5´-GCCCTTCGTGAGAATTGGCT-3´(forward)  5´-AAGCCCTCTTCAGCTTGTGTT-3´(reverse)  5´-ACAATTTGCTAGGCACCCCA-3´(forward)  5´-TCATCTGAAGAGTCTCCTCCTCT-3´(reverse)  5´-ATCTTTCTCCACGTTCGCGT-3´(forward)  5´-GTCTCTCTCCGAGGTAGGGG-3´(reverse)  5´-ATGATGGCAGGTCCTCTGTT-3´(forward)  5´-CCAGCCTAGACACAACTCCTG-3´(reverse) |

**Supplementary Table 3. siRNA Sequence**

| siRNA | Antisense (5´-3´) |
| --- | --- |
| si-RLIM#1 | CCGAUUGGAUCGAGAAGAATT UUCUUCUCGAUCCAAUCGGTT |
| si-RLIM#2  si-RLIM#3  si-MDM2#1  si-MDM2#2  si-MDM2#3  si-TRIM36#1  si-TRIM36#2  si-TRIM36#3 | CCAUCUGCAAGACGUUCUATT UAGAACGUCUUGCAGAUGGTT  CCUUGAUCUUCAAGUAAGATT UCUUACUUGAAGAUCAAGGTT  CCUACUGAUGGUGCUGUAATT UUACAGCACCAUCAGUAGGTT  GGAAAUGCACUUCAUGCAATT UUGCAUGAAGUGCAUUUCCTT  GGACCUUGUACAAGAGCUUTT AAGCUCUUGUACAAGGUCCTT  GAGUAGAGCUGGAUUUAAUTT AUUAAAUCCAGCUCUACUCTT  GUCACCUACUUCUUCUAAUTT AUUAGAAGAAGUAGGUGACTT  GAGCUUACAAGGGUUCAAUTT AUUGAACCCUUGUAAGCUCTT |
